# The New Phases due to Symmetry Protected Piecewise Berry Phases; Enhanced Pumping and Non-reciprocity in Trimer Lattices

**DOI:** 10.1038/srep45015

**Published:** 2017-03-24

**Authors:** Xuele Liu, G. S. Agarwal

**Affiliations:** 1 120 W Miller Ave, Stillwater, Oklahoma 74078, USA; 2 Institute for Quantum Science and Engineering, Department of Biological and Agricultural Engineering, Texas A&M University, College Station, TX 77845, USA.; 3 The Department of Physics, Oklahoma State University, Stillwater, Oklahoma 74078, USA.

## Abstract

Finding new phase of matter is a fundamental task in physics. Generally, various phases or states of matter (for instance solid/liquid/gas phases) have different symmetries, the phase transitions among them can be explained by Landau’s symmetry breaking theory. The topological phases discovered in recent years show that different phases may have the same symmetry. The different topological phases are characterized by different integer values of the Berry phases. By studying one dimensional (1D) trimer lattices we report new phases beyond topological phases. The new phases that we find are characterized by piecewise continuous Berry phases with the discontinuity occurring at the transition point. With time-dependent changes in trimer lattices, we can generate two dimensional (2D) phases, which are characterized by the Berry phase of half period. This half-period Berry phase changes smoothly within one state of the system while changes discontinuously at the transition point. We further demonstrate the existence of adiabatic pumping for each phase and gain assisted enhanced pumping. The non reciprocity of the pumping process makes the system a good optical diode.

The discovery of the Berry phase^1^,^2^,^3^ and the theory of Quantum Hall effect^4^,^5^, have led to large number of studies on the topological states of matter. Three distinct properties characterize non-interacting topological states of matter. These are the Berry phase, discrete symmetry and band gap between the energy bands in parameter space. In translationally invariant systems, Bloch momentum **k** is the parameter and the Brillouin zone is the parameter space. The Berry phase is then the external phase acquired by the eigenstate *ψ*_*n*_ (**k**) of Hamiltonian 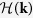 while parameter **k** changes adiabatically around a loop in the Brillouin zone. For electrons, the physics is determined by the filled energy bands. A *characteristic parameter* can be defined based on the Berry phases of the filled bands^4^,^5^,^6^,^7^,^8^,^9^,^10^. The discrete symmetry of system allows only discrete values of the characteristic parameter^6^,^7^,^8^,^9^. Each discrete value relates to a specific topological structure (a complete ball, a complete torus, etc.) and is called the topological number^11^. Continuous changes of parameters of the Hamiltonian may continuously deform the energy bands, however it can not change the characteristic parameter, unless the band gap closes and reopens to form a new type of band structure^11^,^12^. Therefore different matter states are labeled by the discrete topological numbers. These topological phases have distinct physical properties such as type and number of robust edge modes and the corresponding quantum electric transport^13^,^14^,^15^. Any perturbation with respect to the symmetry which preserves the band gap can not destroy the phase^16^. The symmetry is important as when it is broken, the system can then change from one phase to another without closing the gap^17^.

Note that for the quasi-1D system, each filled energy band *ε*_*i*_(*k*) has the Berry phase (module 2*π*) 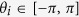. The quantity 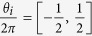 gives the position of the Wannier center of the corresponding energy band, i.e. the center of mass of electrons in each unit cell assuming that the size of the unit cell to be unity^9^,^18^,^19^,^20^. A non-zero Berry phase 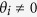 means the center of mass of electrons is not same as the center of mass of atoms, the system is then said to be polarized. For 1D sub-lattice system^6^,^7^,^8^, sum of Berry phases of filled bands is a characteristic parameter. Non-trivial topology of such a system only allows *θ*_*i*_ = ±*π*, leading to maximum non-zero polarization of the system. When the system is finite i.e. has boundaries, the polarization is reflected by the occurrence of extra edge modes eigenstates, for which electrons are localized at the boundary^14^. The 2D nontrivial topology leads to an important new aspect which is called adiabatic pumping^21^,^22^. This will be discussed at length in Secs IV and V.

Thus to summarize the most important aspects of the 1D and the 2D topology are the Berry Phase connection to the topological numbers and the adiabatic pumping. In this article we present our theoretical results on trimer 1D lattices. These lattices deviate from the standard topological results. For example here one gets phases [states of the matter] characterized by piecewise discontinuous Berry phases rather than discrete Berry phases. However such lattices do retain many of the aspects of the adiabatic pumping and edge modes for finite lattices. We specifically consider photonic realization of the trimer lattices. The photonic realization consists of coupled waveguides. The coupled waveguide systems are equivalent to lattices as described in this work. It should be borne in mind that these are different from optical lattices which one makes using standing waves in a system of ultracold atoms. Such waveguide structures are now routinely written on a chip by using femtosecond lasers^23^,^24^,^25^,^26^,^27^. The waveguide arrays are fabricated by using the femtosecond laser micromachining technology. Here we use the nonlinear absorption from an intense focused beam to permanently change the refractive index in localized regions of a transparent material like fused silica. The waveguide pattern is written by moving the sample relative to laser beam in a desired path with a uniform velocity. The 3D precision translation of the sample allows almost any pattern of the waveguide can be written. The change in refractive index depends on the energy in focal volume and therefore the refractive index of a waveguide can be changed by changing the velocity of the sample. The coupling constant among the different units of the unit cell can be adjusted by changing the distance between two waveguides. The technique described here is quite versatile and hence fabrication of trimer lattices should not be a problem. We show how the bending of waveguides can be used to bring additional dimensionality to the system and thus various aspects of the 2D phases can be studied. We discuss the new phases [states] that can arise due to the existence of the symmetry of the unit cell. We will denote it by UCS - as we discuss later - it sets a constraint on the hopping strengths.

Our key findings are— 1. the existence of piecewise continuous Berry phases which define two new 1D phases with the phase transition occurring at the discontinuity of the Berry phase; 2. existence of edge modes localized at the opposite edges for the two different phases [states] and the tomography of such modes; 3. The 2D realization using 1D lattice of trimer leads to phases [states] characterized by very specific 2D Berry phases of half period, these characteristic Berry phases change smoothly within a phase [state] while change discontinuously at the transition point; 4. The existence of adiabatic pumping for each phase; 5. Existence of gain assisted enhanced pumping; 6. Non-reciprocity of the pumping process making the system a good optical diode. The origin of non-reciprocity in our linear device is traced to certain symmetry properties. This is distinct from recent approaches based on nonlinear optical methods^26^,^28^,^29^,^30^,^31^,^32^. The addition of gain and loss is especially important for utilizing edge modes for the pumping and the nonreciprocal behavior of the system.

## Results

### New phases of one dimensional systems given by piecewise Berry phases

**Figure 1:**
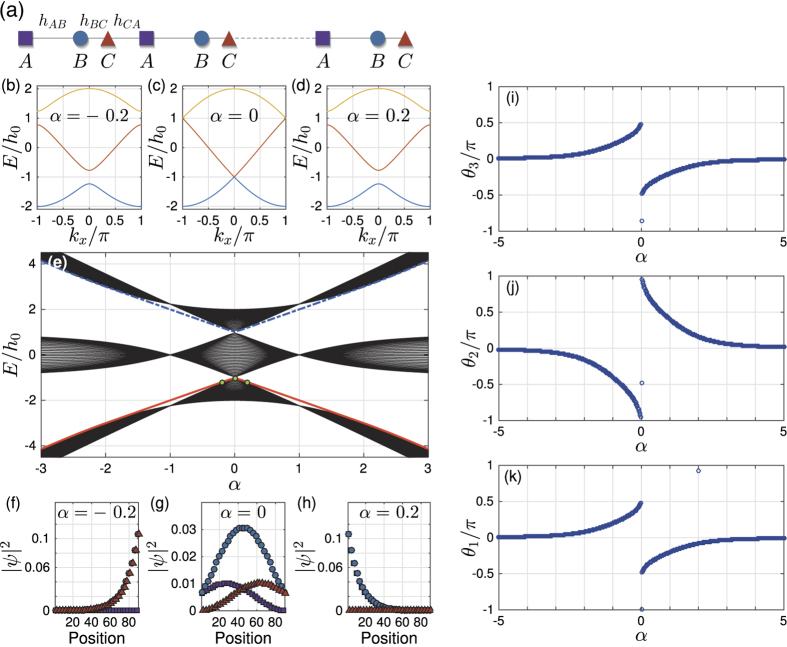
(**a**) Schematic picture of trimer lattices, each unit cell contains three atoms. (**b**–**d**) Spectrum of 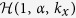 (see equation (3)) as the function of 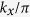. (**e**) Spectrum of the corresponding finite trimer lattice *H*(1, *α, W*) as a function of *α*. The width of sample is *W* = 30 complete unit cells, a.k.a. 90 sites. Red solid line and blue dashed line show the edge modes. The parts (**f**–**h**) gives the corresponding occupation probabilities at the three green dots of the subplot Fig. 1e: purple square, distribution at sites *A*; blue dot, distribution at sites *B*; red triangle, distribution at sites *C*. The parts (**i**–**k**) gives the Berry phases of the three energy bands as the function of *α*. (**i**), the top band; (**j**), middle band; (**k**), the bottom band.

Our investigations are based on a 1D trimer lattice where each unit cell consists of three sites with a specific form of symmetry to be referred to as the unit-cell symmetry (UCS in short). The fundamental eigenvalue equations for a trimer lattice are given by


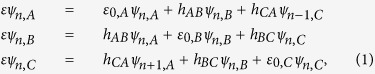


here *ε* is the eigen energy, *n* is the index of unit cell, *A, B, C* label the three different sites in each unit cell (Fig. 1a), *h*_*AB*_, *h*_*BC*_, *h*_*CA*_ are the coupling between the two sites, they are real; *ε*_0,*A*_, *ε*_0,*B*_, *ε*_0,*C*_ are the on-site energies which we assume to be real and equal. As non-zero values of the on site energies give over-all energy shift, we can set these as zero. However positive or negative imaginary parts of on-site energies are used in the latter discussion. This inclusion produces important new results specifically in the context of pumping. With the same on-site energies, UCS is given by the constraint on the coupling 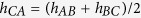. Now we will show that UCS makes the Berry phase piecewise continuous rather than a discrete number and can bring two new phases of matter. The UCS is an important symmetry very relevant to trimer lattices only. We note that the dimer lattices have been well discussed^33^. In this case the chains (*A* = *B* − *A* = *B*−) and (*B* − *A* = *B* − *A*=) are two different phases. At the boundary between the two phases, we may have edge modes. An optical realization is discussed in ref. 34 where by adding gain and loss terms, one obtains non-decaying edge mode and decaying bulk modes. This work also addresses the enhanced pumping due to non-decaying edge modes.

For the trimer lattice of infinite length, the system is translationally invariant and can be described by the Bloch Hamiltonian


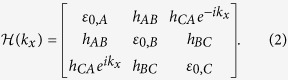


The Berry phase of the 1D trimer lattice can be calculated by this translationally invariant Hamiltonian. With *ε*_0,*A*_ = *ε*_0,*B*_ = *ε*_0,*C*_ = 0, and 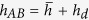 and 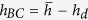, the Hamiltonian can be simplified as


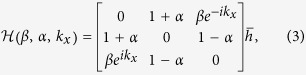


where 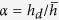, and the constraint 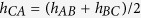 is given by 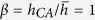. It is convenient to choose the overall factor 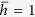. For *β* = 1, i.e. when the system posses UCS, the eigen problem of 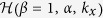 can be solved numerically. For each eigenstate 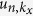 (*n* = 1, 2, 3), we can obtain the Berry-Wilczek-Zee connection 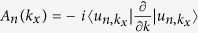
2,3, and calculate the corresponding Berry phase 
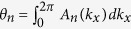
. By setting 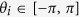, the freedom of the Berry phase is fixed, and has the physical meaning of center of mass of electrons within one cell.

With the new parameters *α* and *β*, Hamiltonian of trimer lattice of finite length *W*, equation (1) can also be rewritten as *H(β, α, W*). For convenience we only consider *β* > 0. However, it is easy to generalize to *β* < 0. Note that since 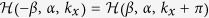, the Berry phase of 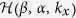 is same as of 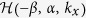; Numerical calculations show that the spectra of *H*(±*β, α, W*) are same and the corresponding eigenstates only differ in phase.

All the numerical results are shown in Fig. 1. The subplots Fig. 1b–d give the energy bands of 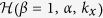. The band gaps are closed at *α* = 0 and open when *α* > 0 or *α* < 0. The Fig. 1e gives the spectrum of trimer lattice *H(β* = 1, *α, W*) of finite length *W*, i.e. the translational invariance is broken and *k*_*x*_ is no more a good quantum number. The edge modes occur for the finite trimer lattice, they are localized at the left edge for *α* > 0 (Fig. 1f) and at the right edge for *α* < 0 (Fig. 1h).

From the distinct behavior of eigenvalues and eigenstates, it is clear that *α* > 0 and *α* < 0 are two different phases separated by *α* = 0. We will now characterize these states via the Berry phases of the eigenfunctions of 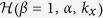. We can see that Berry phases of all the three bands are discontinuous at *α* = 0 (Fig. 1i–k). For the lowest band and the top band, we have *θ* = −*π*/2 at 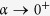 and *θ* = +*π*/2 at *α* → 0^−^. The Berry phase *θ* decreases when |*α*| increases. For the multi-electron ground state that only the lowest band is filled, this picture means when the system is at the ground state, it is negatively polarized for *α* > 0 and positively polarized for *α* < 0, and the polarization reaches maximum at |*α*| → 0. Thus these two are physically distinct phases and disconnect with each other unless the band gap closes.

Note that *α* = 0, *β* = 1 means that the hopping terms between any two sites are same. The discontinuity of Berry phase means *α* = 0 is not stable. Any small disorder may make the lattice positively polarized or negatively polarized. The instability at *α* = 0 is in fact the instability of the 1D lattice that is composed of the same sites.

**Figure 2:**
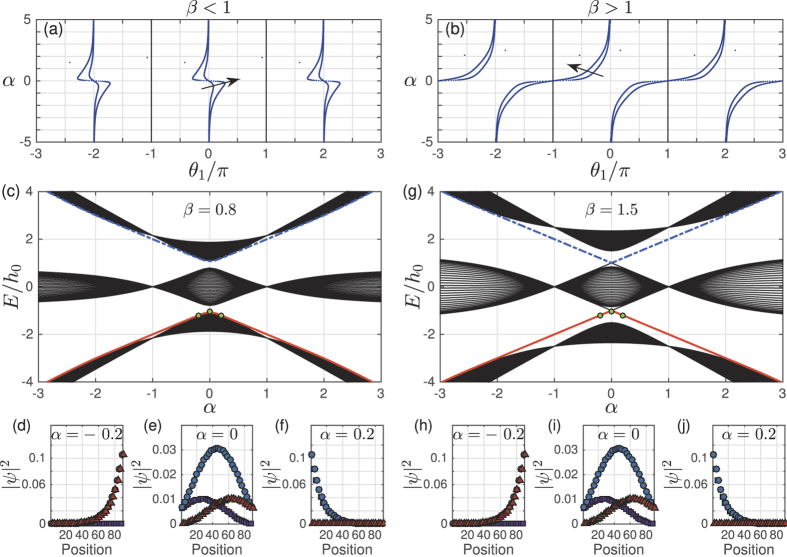
Berry phases, spectrum and eigen states of 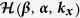 (equation (3)) for *β*≠1: left plots: *β* < 1, right plots: *β* > 1. Plots (**a**,**b**) give evolution of Wannier center (Berry phase) *θ*_1_/*π* of the lowest band while *α* changes, three unit cells are shown (Compare to Fig. 1i–k, the x, y-coordinates are exchanged). Each plot contains two different *β*, along the direction of arrow: (**a**) *β* = 0.5, 0.9; (**b**) *β* = 1.1, 1.5. The plots (**c**,**g**) show spectrum of finite trimer lattice *H(β, α, W*) as the function of *α* for different *β*. The width of sample is *W* = 30 unit cells. The parts (**d**–**f**,**h**–**j**) give the corresponding occupation probabilities at the position of three green dots of the two subplots (**c**,**g**) separately.

Now we may discuss the symmetry of the system. It is easy to check that the Hamiltonian (3) has the inversion symmetry 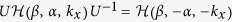, with the inversion matrix 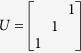. As Berry phase is the center of mass of electrons, the inversion symmetry means center of mass of electrons is also inverted, i.e. *θ(α*) = −*θ*(−*α*). However, this symmetry can not guarantee the discontinuity at *α* = 0. The discontinuity is due to the UCS 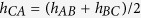 or *β* = 1. This can be seen from Fig. 2. The subplots Fig. 2a,b clearly show that for both *β* > 1 and *β* < 1, the Berry phases are continuous at *α* = 0. Compare them to Fig. 1i–k, it is clear that UCS *β* = 1 guarantees two distinct phases for *α* > 0 and *α* < 0. On comparison, when UCS *β* = 1 is broken, the system can be continuously tuned from the *α*^+^ phase to the *α*^−^ phase without closing the band gap (see Fig. 2c,g). Thus the constraint *β* = 1 has the same role as the symmetry on the non-trivial topological system, though no global unitary symmetry matrix 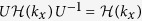 can be found. Since it is the constraint on the unit-cell, we call this constraint as unit-cell symmetry (UCS).

**Figure 3:**
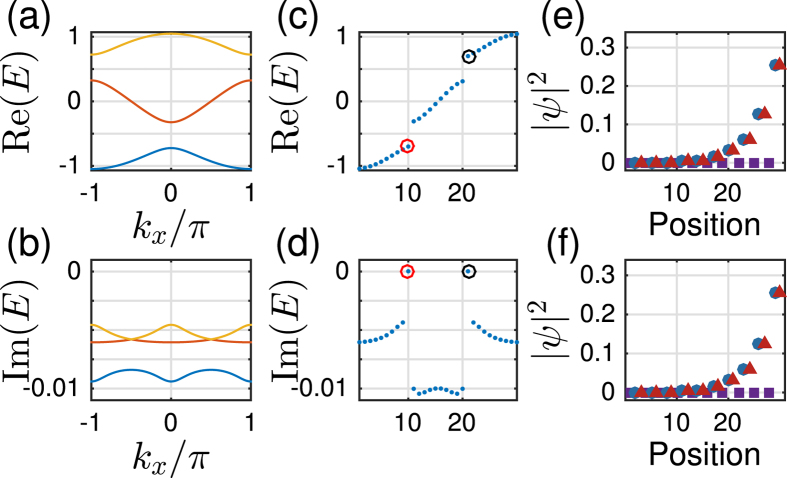
(**a**,**b**) For infinite complex trimer lattice, real and imaginary spectrum as the function of dimentionless wavenumber *k*_*x*_/*π*; (**c**,**d**) real and imaginary spectrum of a finite trimer lattice which contains *W* = 10 unit cells. The x-coordinates are the index of the energies, which are ordered by the real parts. Two extra edge modes are marked by red (*E*_1_) and blue (*E*_2_) circles; (**e**,**f**) distributions of *E*_1_, *E*_2_.

We next return to the question of edge and bulk modes for the model, we will show the edge modes are robust and distinct from bulk modes even when the system is open to the environment. For equation (1), we choose the coupling between sites *h*_*AB*_ = 0.35*h*_0_, *h*_*BC*_ = 0.7*h*_0_ and *h*_*CA*_ = 0.5*h*_0_, i.e. *α* = −1/3 and 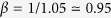 in equation (3). Thus we look at the *α*^−^ phase. In addition, we add a small imaginary part to the on-site energies, specifically we choose *ε*_0,*A*_ = −0.02*h*_0_*i, ε*_0,*B*_ = 0.02*h*_0_*i* and *ε*_0,*C*_ = −0.02*h*_0_*i*, i.e. sites *A* and *C* have the same loss, but site *B* has gain. We match the loss and gain rates. Here the overall scaling factor *h*_0_ is chosen as unity. The plots in Fig. 3 show a remarkable result: the edge modes do not decay while the bulk states decay. The spectrum for infinite lattice (equation (2)) is given by Fig. 3a,b. Compared to infinite lattice, the trimer lattice of finite length (equation (1)) contains two extra modes which are the edge modes. The real parts of eigen energies of these two edge modes are in the real gap between bands (Fig. 3c). The imaginary parts of eigen energies of edge modes are exactly zero (Fig. 3d). In contrast, the imaginary part of the normal eigen modes is always smaller than zero. This can be clearly seen from Fig. 3d or by comparing with Fig. 3b. The distribution of the two edge modes is almost same and is localized at the right edge of the lattice (Fig. 3e,f). We may also find that the distributions at sites *B* and *C* are same and there is almost no distribution at site *A*. The distributions decay fast from the edge, roughly at rate 
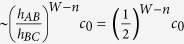
, here *n* is the index of the unit cell, *W* is the total width (i.e. total number of unit cells), *c*_0_ is the probability at the right most side. Thus the two extra modes are called edge modes, and other modes are called bulk modes. For such a system the propagation of light can be used to do tomography of the non-decaying edge modes as shown in Section IV. This is because the bulk modes decay away. It should be mentioned, the zero decay of edge modes is due to the way we choose the imaginary part of the on-site energies. However, even if we choose the imaginary part in a different way so that edge modes also decay, both the real and imaginary spectrum of edge modes are still away from bulk modes and the distributions are still localized, which make them physically distinct from bulk modes.

### New phases of the two dimensional systems characterized by the piecewise half-period 2D Berry phases

For the 1D system, Fig. 2 shows that the two phases *α* > 0 and *α* < 0 are no more distinct when 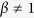. It also shows that, *β* > 1 and *β* < 1 are the two different ways to break the UCS *β* = 1. As the physical meaning of Berry phase *θ*_1_/2*π* is the center of mass of electron within the unit cell, Fig. 2a,b shows the average motion of electron while *α* changes, three connected cells are shown^9^. For 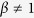, the two disconnected piecewise Berry phases (Fig. 1i–k) are now smoothly connected at *α* = 0, which means the electrons can smoothly move from positive position to the negative position. However, the motions for *β* > 1 and *β* < 1 are totally different. For *β* < 1, two pieces of Berry phases are connected at *θ*_1_ = 0, the electron can only moves within one cell (Fig. 2a). For *β* > 1, two pieces of Berry phases are connected at the cell boundary *θ*_1_ = ±*π*, the electron can move from one cell to another (Fig. 2b).

We may effectively build 2D system by smoothly connecting the two 1D phases *α* > 0 and *α* < 0 with the Hamiltonian,


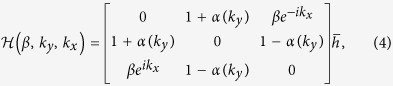


with the periodic term *α(k*_*y*_) = *α*_0_ cos *k*_*y*_. From the inversion symmetry of the 1D system (3), it is easy to check that the Hamiltonian (4) has the inversion symmetry 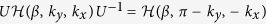 and correspondingly *θ(k*_*y*_) = −*θ(π* − *k*_*y*_). The inversion-symmetric topological insulators have been well discussed for even-band systems^9^,^35^. Here for triple-band system, we show that the cases *β* > 1 and *β* < 1 are two distinct 2D phases although they are not the topological system in the usual sense. The discussion in the following supposes that only the lowest band is filled, thus the edge modes between the top and middle bands do not participate.

**Figure 4:**
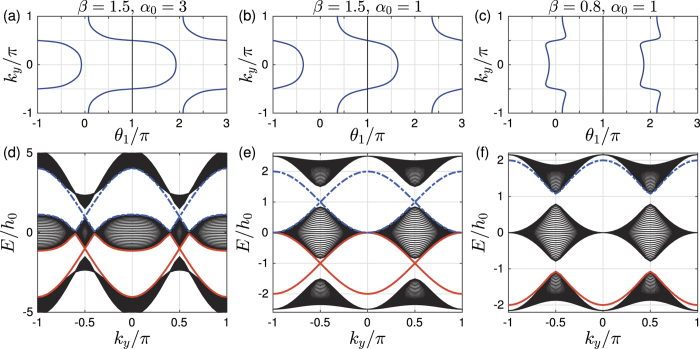
(**a**–**c**) Wannier center flow of the lowest band of 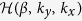 (equation (4)) for different *α*_0_ and *β*; (**d**–**f**) The spectrum of the corresponding quasi-1D *H(β, k*_*y*_) as a function of *k*_*y*_. The width of sample in *x* direction is *W* = 30 unit cells. The sample is infinite in *y* direction so that *k*_*y*_ is a good quantum number. Red solid line and blue dashed line give the two edge bands.

For a translationally invariant system, non-zero integer numbers of 2D Berry phase 
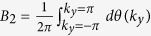
 are used to characterize the non-trivial 2D 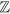 topology, with *θ(k*_*y*_) the effective 1D Berry phase of the filled band for fixed *k*_*y*_. However, for our model, we need some other characteristic parameter to characterize phases. This is because *B*_2_ for equation (4), with the choice *α(k*_*y*_) = *α*_0_ cos *k*_*y*_, is always zero as *θ*_1_(*k*_*y*_) = *θ*_1_(−*k*_*y*_), the two half periods cancel each other (Fig. 4a–c). It is useful to introduce the Berry phase of half period (*α(k*_*y*_) changes from −*α*_0_ to *α*_0_) 
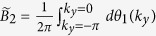
 which is nonzero.

Let us first examine the details for the case *β* > 1. It is clear that when |*α*_0_| → ∞, the behavior of the half period Berry phase is like a Chern insulator, as 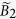 is an integer. In particular, with the integer number 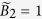 as *θ*_1_ smoothly goes from 0 to 2*π* in the half period −*π* ≤ *k*_*y*_ ≤ 0 and −*α*_0_ ≤ *α* ≤ *α*_0_ with |*α*_0_| → ∞. This behavior can be seen from Fig. 2b for *α*_0_ ~ 5. Because *θ*_1_(*k*_*y*_) interpolates across the maximal possible range [−*π, π*] within one *k*_*y*_ cycle −*π* ≤ *k*_*y*_ ≤ *π*^9^, non-trivial 2D 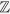 topology can be defined for |*α*_0_| → ∞. When the system is finite in *x*-direction, Fig. 2g shows that the edge mode bands in the bulk-band gap smoothly connect the two bulk bands. This connection makes the system ‘gapless’, and these edge modes are called gapless edge mode, which are believed to be the characteristic behavior of the non-trivial topology.

However, if |*α*_0_| is finite, *α*_0_ cos *k*_*y*_ changes from −*α*_0_ to *α*_0_, the tails of the 1D Berry phase *θ*_1_ in Fig. 2b for |*α*| > |*α*_0_| are not included in the integral of 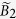, we should have 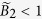. For one specific *β*, the tail is determined by 1D Berry phase 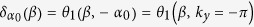, it can be shown that 
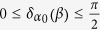
 (see Figs 1j, 2a,b or 4a–c). Due to the inversion symmetry, we may find 
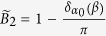
. Thus for finite |*α*_0_|, the system is no more topologically non-trivial as 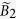 is no more an integer. However, it is still possible to get the gapless edge modes which connect the two bands when |*α*_0_| is large enough (Fig. 4d with |*α*_0_| = 3), thus the occurrence of the gapless edge mode is not the characteristic behavior of topological states of matter, it can occur even when 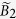 is non-integer. On the other hand, the gapless edge modes are not necessary for *β* > 1. If |*α*_0_| is small, for example |*α*_0_| = 1 (Fig. 4e), the edge modes will not connect to the bottom band. We can smoothly change |*α*_0_| from infinite to any finite value without closing the gap (Fig. 2g). This means that all of them should belong to the same phase of *β* > 1. Discrete characteristic number, gapless edge modes are no more the signatures of the phase. Instead, the characteristic behavior of the phase is that the edge modes must connect to the middle band (Fig. 4d,e). A consequence of it is a measurable quantized Hall conductance. We can also conclude that, the phase for *β* > 1 implies that the center of mass of electron oscillates at the boundary of two unit cells.

In contrast, For *β* < 1, the center of mass of electron oscillates around the center of unit cell (Fig. 4c). The half period Berry phase is given by 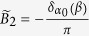 with 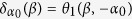. Now, the edge modes are within the bottom band, and directly connect to it at *α*_0_ cos *k*_*y*_ = 0. Thus no edge modes occur in the gap (Fig. 4f). The Hall conductance purely due to the edge modes is hard to get.

Another difference between the phases for *β* > 1 and *β* < 1 is the asymptotic behavior, which can be seen from Fig. 2a,b. For |*α*_0_| → ∞, the oscillation of center of mass of electron is pronounced for *β* > 1 while is negligible for *β* < 1. In contrast, for |*α*_0_| → 0, the oscillation of center of mass of electron is relatively small for *β* > 1 while is pronounced for *β* < 1.

It is clear that *β* > 1 and *β* < 1 are two distinct 2D insulator phases for a fixed value of *α*_0_. The 2D phase is characterized by the half period Berry phase 
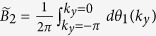
. For *β* = 1 (Fig. 1), within the first Brillouin zone, the gaps of the two energy bands of the 2D material 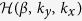 (equation (4)) are closed at *k*_*x*_ = 0, 2*π*, and *α* = *α*_0_ cos *k*_*y*_ = 0, i.e. *k*_*y*_ = *π*/2, 3*π*/2. The gap closing witnesses a phase transition. We find that 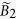 jumps from 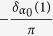 (for *β* < 1) to 
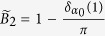
 (for *β* > 1). Here 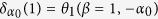 at the gap closing can be directly obtained from Fig. 1k.

The two 2D phases *β* > 1 and *β* < 1 reflect the boundary physics along *x* direction. This is because the difference between the two phases are the oscillation positions of the center of mass of electrons, which depends on the choice of the unit cell along *x* direction^9^. If we choose a new cell by a shift so that the center and the boundary are exchanged, and if the trimer lattice consists of such cells then the physics of two phase is interchanged.

### The pumping process for a 2D lattice

**Figure 5:**
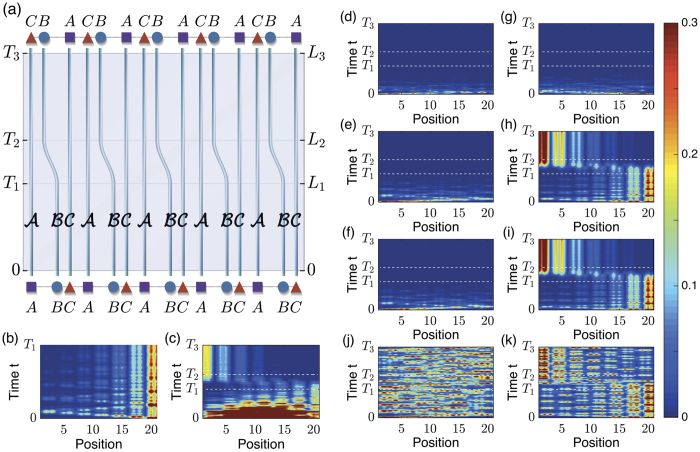
(**a**) Schematic picture of pumping by tuning the hopping rates. The sites of the trimer lattice are marked by *A, B, C*, while the sites of waveguides are marked by calligraphic symbol 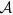, 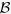, 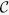. The bottom of waveguides is equivalent to the trimer lattice at *α*^−^ phase while the top is equivalent to the trimer lattice at *α*^+^ phase. The distance between waveguides is chosen as the inverse of hopping, e.x., 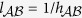. (**b**–**k**) Evolution of lights along the tuned waveguides of (**a**) with different initial states. The width of the sample is *W* = 7 unit cells (21 waveguides). Parameters of the time dependent Hamiltonian *H(t*) are given by equations (6 and 7) and the contexts. (**b**) Only the first time range 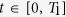 is considered, with *T*_1_ = 500/*h*_0_. We have the tomography of edge modes, with the light fed in from the most right 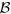. For the rest of subplots (**c**–**k**), the time ranges are chosen: *T*_1_ = 2*T*_0_, *T*_2_ = 3*T*_0_ and *T*_3_ = 5*T*_0_ with *T*_0_ = 200/*h*_0_. (**c**) Evenly feed lights from all the waveguides. (**d**–**f**) Feed in light from the most left unit cell: (**d**) Fed in from the most left site, i.e. the 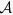 waveguide; (**e**,**f**) Feed in from the most left 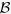, 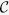 separately. (**g**–**i**) Fed in light from the most right 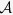, 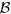, 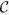 waveguides separately. (**j**,**k**) Without the imaginary part of on-site energies 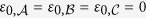: (**j**) From the most left wave guide 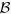; (**k**) From the most right waveguide 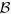.

As mentioned in the beginning, non-trivial 2D topology is demonstrated by adiabatic pumping. However, non-trivial topology is not the necessary condition for pumping. For our system, the half-period Berry phase 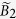 measures number of particles being adiabatically pumped from one edge to another during the half period. Different with topological systems, 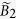 of the two new 2D phases are not integers. As an application, we now discuss pumping in the photonic version of trimer lattices, i.e. we consider an array of waveguides as shown in Fig. 5a^ ^23,24,25,26,27,34. Small imaginary parts are included in the photonic pumping so that we can obtain a clear signal of pumping^34^. This makes the pumping non-adiabatic thus the light pumping is not exactly given by 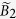.

In Fig. 5a, light fed in from the bottom of waveguides propagates in the three parts in the time range 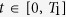, [*T*_1_, *T*_2_] and [*T*_2_, *T*_3_] separately. The time-dependent Hamiltonian *H(t*) (for finite number of unit cell) or 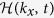 (for infinite number of unit cell) of light has the form equation (1) or (2) with the new time dependent parameters. For example, equation (2) is replaced by


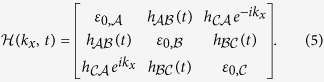


Now the site index *A, B, C* is replaced by the waveguide index 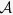, 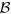, 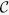. In the following, we check the pumping process for *β* < 1 phase for a half period. The parameters are based on the results of Fig. 3 for the *α*^−^ phase, which also gives decaying bulk modes and satisfy the condition of easy tuning. For both *H(t*) and 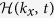, we set 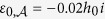, 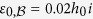, 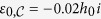, i.e. we choose on-site energies pure imaginary as we add gain and loss so that the bulk states decay away in the first time range 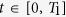; 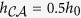 is also fixed for the whole pumping process. The time dependent parameters 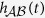 and 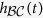 are piecewise functions of *t*: We set *h*_*AB*_ = 0.35*h*_0_ and *h*_*BC*_ = 0.7*h*_0_. We thus have 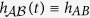 and 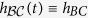 in the range 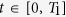; and 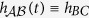 and 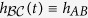 in the range 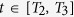. The pumping process in the range 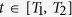 is modeling as follows


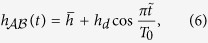



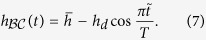


**Figure 6:**
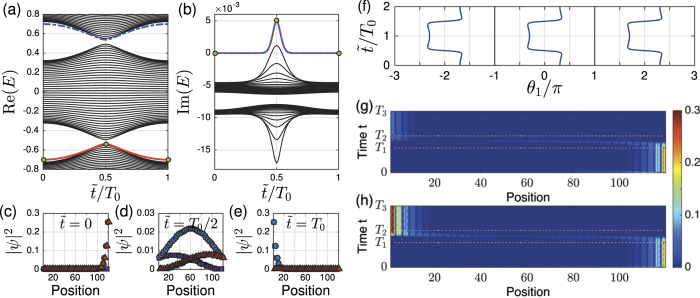
(**a**,**b**) The real and imaginary parts of instantaneous eigen energies *E(t*) of the Hamiltonian *H(t*) as a function of 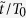 at the tuning range with *T*_0_ = *T*_2_ − *T*_1_ and 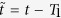. Only a half period 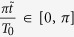 is shown. Here we choose the width of the sample as *W* = 40 unit cells. Other parameters are given around equations (6) and (7). The energies of the two ‘edge’ modes are marked by the blue dashed line and the red solid line. (**c**–**e**) gives the distributions of the red ‘edge’ modes at different time: (**c**) 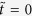 is corresponding to *t* = *T*_1_ of Fig. 5 and (**e**) 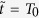 is corresponding to *t* = *T*_2_; (**d**) 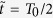 is at the middle of the tuning range. (**f**) The corresponding change of Wannier center *θ*_1_/*π* when 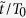 changes. (**g**,**h**) Enhanced pumping due to the behavior around *T*_0_/2. The lights are input from the most right 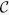 waveguide. For *W* = 40 unit cells, the tuning time are (**g**) 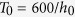; (**h**) 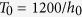.

Here *T*_0_ = *T*_2_ − *T*_1_ and 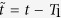. The pumping range 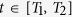 gives 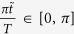, i.e. only the half period is used. We still set 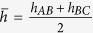 and 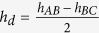. Obviously, 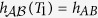 and 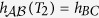. The Hamiltonian for the three time ranges is continuous. The parameters chosen above also guarantee that the band gaps of 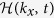 or *H(t*) are not closed for the whole pumping process-see the results in (Fig. 6a,b). In this way we avoid the existence of the exceptional points^36^ of the non-Hermitian hamiltonian, and the system remains diagonalizable^37^. However, it can be checked that pumping process may exist for the gap-closing case.

The propagation of light in such a system can be studied by solving the time dependent Schrödinger Equation 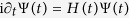. Numerically, we separate the pumping range 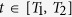 in small time intervals, and suppose at each small interval 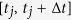, the light propagates with the constant Hamiltonian *H(t*_*j*_). We then obtain


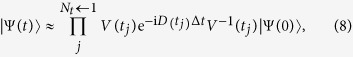



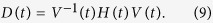


Thus *D(t*) is the diagonalized form of *H(t*) for a given *t*, and the columns of *V(t*) are the corresponding instantaneous eigenstates. Here *N*_*t*_ is number of time intervals, Δ*t* = *T*_3_/*N*_*t*_ is the interval length. 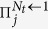 is the abbreviation of *ordered* matrix multiplication. As the matrices do not commute, the matrix with smaller *j* index should be at the right. As long as the time interval Δ*t* is small enough, such a calculation is a good approximation to the time-dependent Schrödinger Equation.

The evolution of light for different initial states is shown in Fig. 5. The initial state depends on how the light is fed in. For example, in Fig. 5c, the light is fed in evenly from all the waveguides, the initial state is then 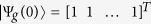. Our plots show the pumping is due to the edge states. In Fig. 5d–f, the light is fed in from the most left unit cell. As initially in the range 0 ~ *T*_1_, there is no edge mode at the left edge (Fig. 3), the light fed in from the left most is carried by the bulk modes, which have completely decayed at the end of the time range 0 ~ *T*_1_. Nothing can be pumped or propagated in the two time ranges *T*_1_ ~ *T*_2_ and *T*_2_ ~ *T*_3_. In Fig. 5g, though the light is fed in from the site of the most right unit cell, it is still totally decays in the range 0 ~ *T*_1_. This is because the right edge states have no distributions at the site *A* (Fig. 3e,f), and the light is thus carried by the bulk modes which decay. The pumping process can be clearly seen from Fig. 5h,i: after a few propagation length in 0 ~ *T*_1_, the small portions of bulk states decay while the edge modes are clearly left at the sites *B, C* (the two bright waveguides on the right). The pumping from right edge to left edge can be seen in time range *T*_1_ ~ *T*_2_. After pumping, in the time range *T*_2_ ~ *T*_3_, the light propagates on the left sides. Here one thing should be mentioned that there is no distribution of light on the waveguide 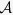 in the time range 0 ~ *T*_1_ and the distribution exists in the time range *T*_2_ ~ *T*_3_. This is because the waveguide 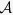 connects site *A* of initial unit cell and site *C* of finite cell, and the edge modes only exist at sites *B, C*. This situation is reversed for waveguide 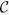. Coming back to Fig. 5c, where the light is evenly fed in from all the waveguides, after long time evolution, at the range *T*_2_ ~ *T*_3_, the distribution is typically like that of edge modes - although in contrast to Fig. 5h,i, there is considerable loss in the output.

We would like to stress that the imaginary parts of the on-site energies 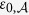, 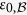, 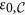 are important to get a transparent and easily visible signal of pumping. This is evident from Fig. 5j,k, the imaginary part of all the on-site energies are set zero, 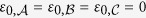 so that the pumping is adiabatic. This change has no remarkable effect on the real part of the spectrum and the eigen states. The only change is that the imaginary parts of all eigenvalues are exactly zero. However, this change changes the pumping considerably. For Fig. 5j, the light is fed in from the most left wave guide 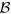, the bulk states give a noisy signal after propagation, whereas in Fig. 5e we have no signal due to decay. For Fig. 5k, the light is fed from the most right waveguide 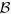, we can see relatively stronger signal at the right edge before pumping and the relatively stronger signal at the left edge after pumping. However, as compared to Fig. 5h, the signals are very noisy due to contributions from bulk states.

### Enhanced pumping

Thus the positive and negative imaginary parts of the on-site energies completely change the nature of pumping by wiping out the noisy contributions of bulk states. Further more, Fig. 5c,h,i show that the light transmission is strengthened during the pumping: the intensities of the left edge modes after pumping are stronger then initial edge modes at the right side.

This enhanced pumping can be well explained by the instantaneous eigen spectrum of the Hamiltonian 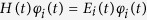. The spectrum *E*_*i*_(*t*) as a function of 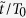 (with *T*_0_ = *T*_2_ − *T*_1_ and 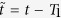) is shown in Fig. 6a,b. Only the half period 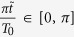 involved in pumping is shown, spectrum of another half period 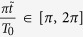 can be easy get from the fact 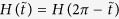 (see equations (6) and (7)). The two edge modes due to the boundaries are marked by blue dashed line and red line. Their real parts lie in the bottom of upper band and the top of the bottom band; The imaginary parts of the two bands overlap and are away from the bulk states. In the full time interval, the two extra modes can not always be treated as edge modes. At 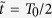, the real parts of edge bands merge into the real bulk bands, the corresponding eigenstates are also non-localized modes (Fig. 6d). This merger leads to some transfer of population from edge modes to bulk modes. The speciality of the small range around 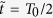 can also be seen from the imaginary part of eigen energies. In most of the time range, the imaginary parts of the two extra bands are zero while the imaginary parts of bulk modes are smaller than zero. However, in the small range around 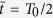 and 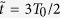, though the imaginary parts of the extra modes are still different from the bulk modes, both the two extra modes and some bulk modes gain energy. The system thus gains energy in this small range. As the imaginary parts of bulk states change to negative after this small period, the energy gain by the bulk states decays to the environment soon. However, as the imaginary part of the two extra modes now becomes zero, the energy gained by the extra modes is retained.

In summary a competing mechanism is introduced in the small range of merging process around 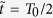: the extra modes may lose energy to the bulk states, which decays away in the following process; it may also gain energy from the gain medium. Our numerical results show that the signal of the output left edge mode can be enhanced if the tuning range *T*_1_ ~ *T*_2_ is made long enough. In such a case, the extra modes can stay at the small range around *T*_0_/2 long enough, so that the energy gain from the gain medium can be bigger then the energy loss to the bulk states. This can be clearly seen for a wide sample. In Fig. 6g, we choose *T*_0_ = 600/*h*_0_, the power gained is not strong enough to fully send the edge mode from left to right, the strength of output left edge modes are weaker then the input right edge modes; In Fig. 6h, we double the tuning range so that *T*_0_ = 1200/*h*_0_, output left edge modes are much brighter then the input right edge modes. Through this way, we obtain enhanced pumping.

### Non-reciprocity in light propagation

**Figure 7:**
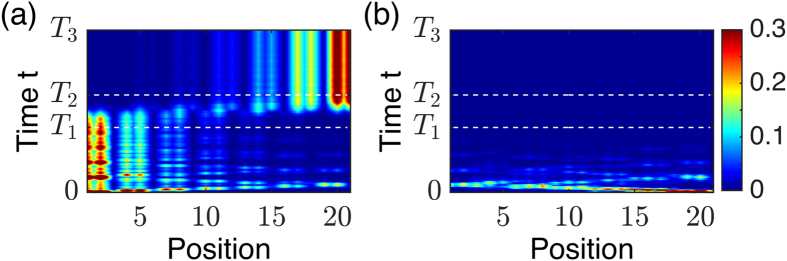
Non-reciprocal property of light pumping. The waveguides set (of Fig. 5a) are up-side down so that *α*^+^ phase is at the bottom and the *α*^−^ phase is at the top. Light is fed in from the *α*^+^ phase: (**a**) From the most left wave guide 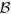; (**b**) From the most right waveguide 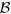. The non-reciprocity is clearly seen by comparing Fig. 7a,b with Fig. 5e,h.

Our trimer lattice exhibits a very important property namely non-reciprocity in propagation. More specifically, the pumping process between two reversed phase *α*^−^ and *α*^+^ is non-reciprocal. In Fig. 7, the waveguides set of Fig. 5a is inverted ie made upside down and the light is fed in from the *α*^+^ phase. In Fig. 7a, the light is fed in from most left 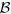 wave guide, we can witness the pumping of light from the left to the right. However, as compared to Fig. 5e, the light fed in the same waveguide from *α*^−^ phase can not be pumped. The same situation happens for input light from the most right waveguide: there is the pumping from the right to the left in the Fig. 5h when the light is input from *α*^−^ phase; while the light fast decays if the light is input from the *α*^+^ phase (Fig. 7b). The non-reciprocal property is due to breaking of the vertical inversion symmetry *P*_*y*_, which is also equivalent to the broken time reversal symmetry. However, the system still has the *π* rotation symmetry or equivalently the combination of the vertical and the horizontal inversion symmetries *P*_*x*_*P*_*y*_, which makes Fig. 7a equivalent to Fig. 5h, and Fig. 7b is equivalent to Fig. 5e after left-right reflection. It should be noted that we produce non-reciprocity using a linear system based on trimer lattices described by equation (1). This is quite distinct from several other recent approaches based on nonlinear optical methods^26^,^28^,^29^,^30^,^31^,^32^.

## Discussion

We first note that all the previous work on topological effects using waveguide systems have been on dimer lattices. Existence of topologically protected mid gap states was predicted in ref. 34. The refs 38 and 39 present experimental results on topologically protected states using an interface between dimer chains. The waveguide systems are extensively used in a number of fields of optics. The trimer lattices have so far attracted no attention though such lattices especially the finite ones exhibit a variety of phases. Here we have presented a detailed study of the new phases which can arise in trimer lattices. We specifically emphasize the new phases occurring in finite systems. By studying 1D trimer lattices we reported edge modes and new phases characterized by Berry phases which are piecewise continuous rather than discrete numbers as in case of topological phases. The phase transition occurs at the discontinuity point. We discussed how trimer lattices can be used to obtain a 2D realization with phases characterized by very specific 2D Berry phases of half period. These characteristic Berry phases change smoothly within a phase while change discontinuously at the transition point. We further demonstrated the existence of adiabatic pumping for each phase and gain assisted enhanced pumping. The non-reciprocity of the pumping process makes the system a good optical diode. The results apply to both electron and photon transport. As discussed in text it is easier to realize the photon transport by using a system of waveguides. We also note that the bending of the waveguides has been demonstrated in ref. 39. We specifically took advantage of adding gain and loss in the waveguides. Clearly the photonic lattices provide a new platform for the study of the phases of matter and enhanced pumping. The phases of trimer lattices are different from standard topological phases. The trimer lattices also allow the non reciprocal propagation and diode action. The trimer lattices with defects are expected to yield much richer physics.

## Additional Information

**How to cite this article:** Liu, X. and Agarwal, G. S. The New Phases due to Symmetry Protected Piecewise Berry Phases; Enhanced Pumping and Non-reciprocity in Trimer Lattices. *Sci. Rep.* **7**, 45015; doi: 10.1038/srep45015 (2017).

**Publisher's note:** Springer Nature remains neutral with regard to jurisdictional claims in published maps and institutional affiliations.
